# The Quantitation of Squalene and Squalane in Bronchoalveolar Lavage Fluid Using Gas Chromatography Mass Spectrometry

**DOI:** 10.3389/fchem.2022.874373

**Published:** 2022-04-07

**Authors:** Elizabeth A. Cowan, Hang Tran, Clifford H. Watson, Benjamin C. Blount, Liza Valentín-Blasini

**Affiliations:** Tobacco and Volatiles Branch, National Center for Environmental Health, Centers for Disease Control and Prevention, Atlanta, GA, United States

**Keywords:** electronic cigarettes, electronic vaping products, bronchoalveolar lavage fluid, gas-chromatography mass spectrometry, squalene, squalane

## Abstract

Chemicals of unknown inhalational toxicity are present in electronic cigarette and vaping products. E-cigarettes typically contain nicotine and other relatively hydrophilic chemicals while vaping products typically contain cannabinoids and other hydrophobic chemicals. For example, vaping products can include hydrophobic terpenes such as squalane (SQA) and squalene (SQE). However, little is known about the SQA and SQE transmission from liquid to aerosol. SQA and SQE are used in commercial products that are applied dermally and ingested orally, but limited information is available on their inhalational exposure and toxicity. We developed and validated a quantitative method to measure SQE and SQA in bronchoalveolar lavage fluid to assess if these chemicals accumulate in lung epithelial lining fluid after inhalation. Calibration curves spanned a range of 0.50–30.0 µg analyte per mL bronchoalveolar lavage fluid. Recoveries were found to be 97–105% for SQE and 81–106% for SQA. Limits of detection were 0.50 μg/ml for both SQE and SQA. The method was applied to bronchoalveolar lavage fluid samples of patients from the 2019 outbreak of e-cigarette, or vaping, product use-associated lung injury (EVALI) and a comparison group. Neither SQA nor SQE was detected above the method LOD for any samples analyzed; conversely, SQA or SQE were reproducibly measured in spiked quality control BAL fluids (relative standards deviations <15% for both analytes). Further applications of this method may help to evaluate the potential toxicity of SQA and SQE chronically inhaled from EVPs.

## Introduction

Use of electronic cigarettes (e-cigarettes), or vaping, products (EVPs) in the United States has increased since e-cigarettes were introduced into the U.S. market (Cullen et al., 2019; Knapp et al., 2019; Meier et al., 2019; Schulenberg et al., 2019; Spindle et al., 2019; Braak et al., 2020). These devices deliver inhalable aerosols that contain active ingredients such as nicotine and cannabinoids. E-cigarettes typically contain nicotine and other relatively hydrophilic chemicals while vaping products typically contain cannabinoids and other hydrophobic chemicals. In a 2018 study of adults using e-cigarettes, 7.1% of participants also report vaping some form of cannabis (Uddin et al., 2020). A more recent study of U.S. youth finds that 19.6% of high school and 4.7% of middle school students reported vaping in the past 30 days (Wang et al., 2020) and 4.2% of 8th graders, 23.5% of 10th graders, 12.2% of 12th graders reported vaping cannabis in the past 30 days (Miech et al., 2021). Furthermore, both adults and adolescents who use cannabis preferred vaping e-liquids (e.g., concentrates from cannabis flower or plant extract) over smoking cannabis (Lee et al., 2016; Morean et al., 2017; Knapp et al., 2019). Multiple reasons could explain the increase in use of both nicotine and cannabinoid delivery devices, including perception of reduced risk compared with smoking and the ease of concealing these potentially-illicit devices. The non-medical cannabis legalization by multiple states and the ease of product customization have likely contributed to the increase in vaping of cannabis (Morean et al., 2017; Cullen et al., 2018; King et al., 2018; Romeh et al., 2018).

Increased demand for EVPs has contributed to a proliferation of sources of EVP liquids, including legitimate vendors, off-market sources, and “DIY” liquids (Kong et al., 2017; King et al., 2018; Romeh et al., 2018). EVP liquids and their components are widely available online and over-the-counter, thus enabling use of unregulated off-market and DIY liquids. Active ingredients are often diluted or “cut” with miscible diluents (hydrophobic or hydrophilic depending on product type) such as polyethylene glycol (PG), vegetable glycerin (VG), medium-chain triglycerides (MCT), and terpene mixtures to increase vaping efficiency, provide aroma and flavors, enhance the appearance of products, and/or to lower product cost (Kosmider et al., 2014; Erythropel et al., 2019).

Squalene (SQE) is a naturally-occurring polyunsaturated triterpene formed by many plant and animal cells as a biochemical precursor of sterols. Topical and oral products have included SQE as an ingredient for decades without reports of adverse health effects (Reddy and Couvreur, 2009; Wu et al., 2016). Squalane (SQA) is the completely saturated form of SQE and is naturally formed by plants and animals (albeit in lesser amounts than SQE). SQA can also be chemically synthesized through hydrogenation of SQE. SQA is more widely used in dermatological products than is SQE, perhaps because of better skin absorption, ability to enhance flexibility of and to moisturize human skin, to act as a carrier to increase absorption of other substances, and its long shelf-life (Reddy and Couvreur, 2009; Wu et al., 2016). Conversely, physical properties such as viscosity, miscibility with other oily substances, and higher boiling point make SQE more likely than SQA to be used as a diluent for hydrophobic EVP liquids.

The U.S. recently experienced an outbreak of e-cigarette, or vaping, products use-associated lung injury (EVALI), with over 2,800 hospitalized cases reported (King et al., 2020; Reagan-Steiner et al., 2020; Werner et al., 2020). Some patients diagnosed with EVALI exhibited respiratory symptoms resembling those previously reported by patients who had aspirated mineral oil (Aberegg et al., 2020; Gay et al., 2020; Shah et al., 2020). In an urgent attempt to identify the cause of the outbreak-associated lung injury, bronchoalveolar lavage (BAL) fluid samples from case patients were analyzed by the Centers for Disease Control and Prevention (CDC) for multiple EVP-associated chemicals (DeJesús et al., 2020a; DeJesús et al., 2020b; Holder et al., 2021; Morel-Espinosa et al., 2021; Xia et al., 2021; Brosius et al., 2022). Limited clinical observations indicate that SQE can cause lipoid pneumonia following accidental inhalation, and thus SQE and SQA were considered potential substances of interest (Lee et al., 1999; Kanaji et al., 2008; Cha et al., 2018).

SQE has been quantified in various matrices including olive oil, human hair, human serum, and incense smoke by means of gas chromatography flame ionization detection and gas chromatography-mass spectrometry (GC-MS) (Wu et al., 2016; Santivanez-Veliz et al., 2017; Budge and Barry, 2019; Pacetti et al., 2019; Aresta et al., 2020). Qualitative confirmation of SQE in human BAL fluid (Kanaji et al., 2008) has been reported using GC-MS. However, quantitative analytical measurements of SQA and SQE have not been previously reported in BAL fluid. Therefore, we rapidly developed and validated a novel, accurate, and precise method for quantifying squalane and squalene in BAL fluid using GC-MS. The intended purpose of this method is to measure SQA and SQE in BAL fluid at concentrations capable of causing lung injury and/or lipoid pneumonia. Therefore, the method was applied to BAL fluids collected from people with EVP-related lung injury and from otherwise healthy comparators.

## Materials and Methods

### Chemicals and Reagents

Reference standards of squalene (≥98% purity) and squalane (≥96% purity) were purchased from Sigma (St. Louis, MO). Cannabinol-D^3^ (CBN-D^3^) 100 μg/ml solution in methanol was purchased from Cerilliant Corp. (Round Rock, Texas). Simulated lung fluid—Gamble’s formulation (not stabilized) was purchased from Pickering Laboratories Inc. (Mountain View, CA). Solvents including hexanes and methanol (Optima for GC) were purchased from Fisher Scientific (Pittsburgh, PA). Research-grade helium (He) and ultra-high purity grade nitrogen (N_2_) gases were obtained from Airgas, Inc. (Hapeville, GA).

### Instrumentation

The BAL fluids were analyzed using an Agilent 7890B GC System with an Agilent Sampler 80 and interfaced Agilent 7000C triple-quadrupole mass spectrometer (Agilent Technologies, Santa Clara, CA). Chromatographic separation was achieved using a HP-5MS UI (30 mm × 0.25 µm x 0.25 mm) capillary column with a 1.2 ml/min flow of helium carrier gas. GC inlet was operated at 250°C in split mode with a 20:1 split ratio. The initial oven temperature was 150°C and was ramped at 40°C/min to 250°C with a 2 min hold, followed by a second ramp of 5°C/min until reaching a maximum temperature of 300°C. Analytes eluted at 7.693 min (CBN-D^3^), 8.05 min (SQA), and 9.89 min (SQE) with a total run time of 14.5 min. The mass spectrometer source and transfer line were maintained at 280°C and quadrupoles temperatures were 150°C. The MS was operated in positive electron ionization (EI) mode and the resulting ions analyzed by single-ion monitoring (SIM) scan mode. Electron multiplier voltage gain factor was 10 for all ions monitored (SQA: 113 m/z (quantitative), 183, 127, 422 m/z (confirmation); SQE: 137 (quantitative), 410, 411 m/z (confirmation); CBN-D^3^ 298 m/z (quantitative), 313, 312 m/z (confirmation). Data acquisition and analysis were conducted using Agilent MassHunter Workstation Software (Agilent Technologies, Santa Clara, CA).

### Standard Solutions Preparation

Neat materials were weighed gravimetrically, and all solutions were made volumetrically in hexane and stored at −70°C. Individual stock solutions of SQA and SQE were prepared and used to prepare a mixture of the two analytes (1.00 ng/ml) and two additional stock dilutions (0.100 and 0.005 ng/ml). Gamble’s solution is a synthetic fluid that represents the interstitial fluid deep within the lungs that is commonly used in particle inhalation effect studies. Eight calibration standards were prepared daily by spiking 0.500 ml of Gamble’s Solution with the appropriate dilution and 25 µl aliquot of internal standard (ISTD) CBN-D^3^ (100 μg/ml methanol, as purchased) (Boisa et al., 2014). Final calibrator concentrations covered a range of 0.5–30 μg/ml.

### Quality Control Material Preparation

Separate sets of reference materials, stock solutions, mixtures, and dilutions were used in all validation experiments and in the preparation of two quality control (QC) spiking pools. These pools were used to spike commercial synthetic BAL fluid (ml) at a low (QCL) and a high (QCH) analyte concentration. Mean concentrations (SQE: 1.27 and 22 μg/ml; SQA: 1.26 and 20 μg/ml) and the 95th (1.96σ) and 99th (2.96σ) percentile control limits for each pool were determined from five runs over five different days. Each QC pool was analyzed in duplicate with each analytical run and the resulting QC data compared to the established control limits to evaluate the validity of analyses using modified Westgard rules (Westgard et al., 1981; Caudill et al., 2008). All solutions were stored at −70°C.

### Bronchoalveolar Lavage Fluid Collection and Storage

BAL fluid was obtained from 55 EVALI patients as part of their clinical treatment. The lavage procedures were not standardized across the multiple institutions that collected samples because the samples were being collected as part of emergency care or diagnosis of ill patients. Residual BAL fluids were sent to CDC as coded specimens with no personally identifiable information. Samples were refrigerated or frozen after collection and shipped on dry ice to CDC. A CDC human-subjects research review panel judged this collection of samples to be a non-research public health response activity.

Additional BAL fluids had previously been collected from 99 non-EVALI comparators ages ranging from 21 to 45 years. These comparators either did not use tobacco products (52), currently exclusively used nicotine-containing e-cigarette products (18), or currently exclusively smoked cigarettes (29) (Tsai et al., 1999; Song et al., 2020). BAL fluid was transported to the CDC laboratory on ice and processed within 30 min upon arrival by centrifugation, to remove l; cellular debris pellets and supernatant were immediately placed in storage at −80°C (Blount et al., 2020). All non-EVALI participants provided written informed consent before participation. The study of healthy comparators was approved by the institutional review board at the Ohio State University, NCT02596685 (Song et al., 2020).

### Bronchoalveolar Lavage Fluid Sample Preparation

For SQA/SQE analysis, 0.500 ml BAL fluid was thawed to room temperature, vortexed, transferred to a 16 × 125 mm disposable culture tube, and spiked with 25 µl CBN-D^3^ ISTD. Two milliliters of hexane were added, and samples were extracted by 360-degree rotation for 30 min. Samples were held at room temperature until the solvent phases separated; subsequently, the hexane layer was transferred to clean 16 × 125 mm centrifugation tubes using an automated liquid handling and solid phase extraction system (Gilson 274). The hexane layer was dried to completion under a stream of nitrogen in a turbo evaporator equipped with a heated water bath. Samples were reconstituted in 200 µl hexane, vortexed, transferred to amber sample vials with glass inserts and added to the GC-MS autosampler tray for analysis. Solvent blanks were injected after every EVALI case patient BAL fluid to confirm no analyte carryover and to help flush residual lipids from the column.

### Quantitation

All SQA, SQE, and CBN-D^3^ data was evaluated for accuracy integration and manually reintegrated if necessary. Quantitation was based on a set of eight calibration standards (0.5–30 μg/ml) prepared in commercial synthetic BAL fluid same as unknown samples with the exception that standards were spiked with known concentrations of SQA and SQE prior to sample preparation. Calibration curves were constructed using quadratic regression of the analyte-to-ITSD response ratio versus known standard concentrations with 1/X weighting. Results were reported in concentration units (ng analyte per ml BAL fluid).

## Method Validation

Method validation parameters: analytical specificity, sensitivity, recovery, precision and repeatability, and storage stability were studied to confirm that the performance characteristics of the method were accurate and fit-for-purpose as described below.

### Method Specificity

Specificity was verified by comparing chromatograms of 10 random human BAL fluids, pooled BAL fluid, and commercial synthetic BAL fluid to those spiked with SQA and SQE (1.5 μg/ml).

### Accuracy

Accuracy was evaluated using spiked recoveries in hexane, matrix (commercial synthetic BAL fluid), and pooled human BAL at two levels (hexane 2.0 and 4.0 μg/ml; matrix 1.0 and 2.0 μg/ml; pooled human BAL 6.0 and 15.0 μl/mg *n* ≥ 3). Recovery was calculated as the ratio of experimental concentration and the nominal spiked concentration and was considered acceptable between 85–115% (80–120% at 3 times LOD).

### Precision

Method precision was evaluated as repeatability and intermediate precision of 10 QC samples at two levels over a period of 5 days. Repeatability was measured and reported as the percent relative standard deviation (%RSD) of within-run duplicate samples and intermediate precision was calculated as the %RSD of the mean of duplicate samples over 5 separate runs.

### Stability

Analyte stability was assessed by two levels of prepared QC samples at room temperature (GC autosampler tray) for 48 h and at −20°C for 13 days. These conditions were chosen to evaluate analyte stability in prepared samples as the samples were queued for GCMS analysis or stored at −20°C. All samples were sequenced for instrument analysis immediately after preparation in this study.

## Method Application

The validated method was used to analyze a total of 182 BAL fluid samples during the CDC response to the 2019 EVALI outbreak. Fifty-five of the analyzed samples were collected from probable or confirmed EVALI patients from 17 different states. Comparator BAL fluids were also collected from 99 volunteers with no major clinical illness.

## Results and Discussion

**FIGURE 1:**
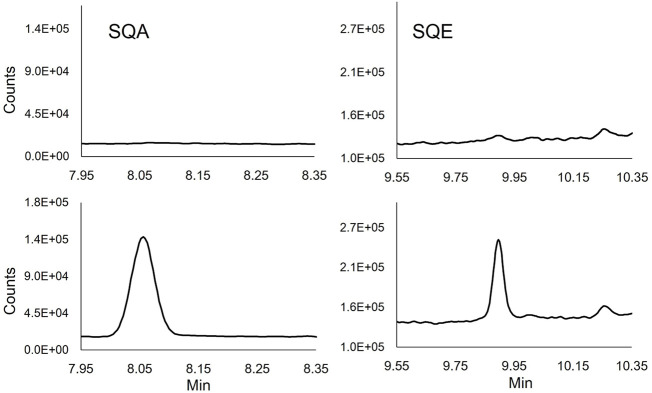
Extracted ion chromatograms of SQE (137 m/z) and SQA (113 m/z) quantifying ions in unmodified (Top chromatograms) and spiked (1.5 μg/ml) human BALF (Lower chromatograms).

The specificity of the method was evaluated in synthetic commercial BAL fluid, pooled human BAL fluids, and 10 individual human BAL fluid samples. The resulting chromatographic data (e.g., baseline interferents, matrix interferents, and peak shape) was compared between samples prepared with only the addition of ISTD and those spiked with relatively low levels of each standard (1.5 μg/ml). No interfering peaks were identified in either the quantitation or confirmation ions for SQE, SQA or ITSD in the pooled and individual BAL fluid samples (Figure 1 and Supplementary Figure S1). No chromatographic peak shape distortions or other visible matrix interferences were observed.

The method was developed to respond to a public health emergency and thus BAL fluid was not readily available to use as a solvent for matrix equivalency experiments. Despite this challenge, validation experiments document that the method performed without significant bias in BAL fluid matrix. Table 1 shows acceptable accuracy following spiking analytes into hexane and synthetic BAL fluid (SQA: 80.6–106%; SQE: 93.2–106%). The lowest calculated recoveries were consistently from SQA spiked into hexane. Additional recovery experiments spiked in pooled human BAL fluid yielded similar results, and all were within the acceptable range (SQA: 91.9–103%; SQE: 83.4–102%). Within-run repeatability was determined to be acceptable with all % RSD being less than 4.5% for all both analytes. Intermediate precision over 5 days was calculated to be below 13% (SQA) and 9% (SQE). Thus, the internal standard was able to produce accurate and precise quantitation in both synthetic and actual BAL fluids despite potential differences in matrix compositions.

**TABLE 1:** Analyte recovery for three fortified matrices (*n* ≥ 3).

Analyte	Matrix	Spike value (µg/ml)	Recovered value Mean (µg/ml)	Recovery (%)	RSD (%)
SQA	Hexane	2	1.94	97.2	1.40
		4	3.22	80.6	1.30
	Synthetic BAL	1	0.860	86.3	11.4
		2	2.12	106	11.2
SQE	Hexane	2	2.16	106	3.80
		4	3.72	93.2	3.30
	Synthetic BAL	1	0.980	97.0	11.3
		2	2.10	105	14.5

The minimum reporting level (MRL) for both SQA and SQE was set at the lowest calibrator concentration, 0.50 μg/ml BAL fluid. This value was verified by evaluating seven separately prepared calibration curves for the lowest calibrator that resulted in a chromatographic peak with a signal-to-noise (S/N) ratio greater than three and a calculated concentration accuracy of ± 40% of the expected concentration. The absence of published methods quantitating these analytes in lung fluid makes it difficult to compare the MRLs reported here to similar methods; the 0.50 μg/ml MRLs are, however, within the range of other analytical methods reporting the detection of SQE in biological samples, such as human hair (0.1 μg/ml), plasma (0.026 μg/ml), and bile (0.104 μg/ml) (Liu et al., 1976; Pacetti et al., 2019).

To ensure analyte stability in prepared samples during the study, a stability study was conducted at room-temperature (21°C in GC autosampler tray) and at −20°C. Both analytes were found to be stable in prepared BAL fluids stored at −20°C for 14 days with low percent changes in analyte concentrations from day 1 to day 14 (<6% decrease for SQA (1.93–5.66%) and less than 11% for SQE (11.30–6.65%). For prepared samples stored at room temperature for 48 h, we observed no differences in measured concentrations*.* Based on these results, SQA and SQE were considered stable in prepared BAL fluids under the conditions tested and were suitable for the intended application of the method; however, a longer stability study is needed to evaluate the stability of the analytes for longer periods of time. Due to the urgency of the results, all case-related BAL fluids were prepared within 24 h of receipt and were analyzed immediately after preparation.

## Application Results

The validated method was applied to analyze SQA and SQE in BAL fluids collected from 55 EVALI case patients and 99 comparators with no EVALI symptoms. Neither the case-related nor the comparator BAL fluids contained SQA or SQE above the method MRL; however, these findings do not indicate that the method was inadequately sensitive. Quality control samples were analyzed in the same analytical batch as unknown BAL fluid samples and yielded expected analyte levels within the stated imprecision of the method (Supplementary Figures S3, S4). Furthermore, the analytical sensitivity of each analysis was confirmed by an acceptable internal standard signal (Supplementary Figure S2). Thus, the effectiveness of the method was confirmed for each batch of analyses (QC quantitation) and for each individual analysis (internal standard signal). The intended purpose of this method was to look for evidence of inhaled SQA and SQE accumulating in BAL fluid to concentrations capable of causing lung injury and/or lipoid pneumonia. Therefore, the focus was on a rapid method that could be developed, validated, and applied to clinical specimens in a matter of weeks. The resulting MRL was 0.5 μg/ml of BAL fluid for both SQA and SQE; Thus, the method is adequately sensitive to detect the level of SQE identified in BAL fluid collected from a patient with lipoid pneumonia caused by SQE aspiration (Kanaji et al., 2008). Another way to evaluate the need for methodological sensitivity is to consider the likely daily inhalation of SQE based on the emissions of EVPs associated with EVALI case patients. EVALI-associated EVPs that contained SQE produced an aerosol with mean SQE concentration of 33 ng/ml puffed air (Cowan et al., 2022). Therefore, an EVALI case patient using one of these SQE-containing EVPs would inhale approximately 27,500 ng SQE for every 15 puffs. By comparison, the method that can detect as little as 0.125 ng of SQE or SQA on column. The finding of no detectable SQE or SQA likely indicates that these chemicals are being cleared from the lungs. Lastly, the method is able to detect SQE and SQA at much lower concentrations than the levels of vitamin E acetate that are attributed to be the primary cause of EVALI (Bhat et al., 2020; Blount et al., 2020; Morel-Espinosa et al., 2021)

Our findings clearly show no measurable accumulation of SQA or SQE in BAL fluids collected from EVALI case patients. These results do not preclude harm caused by vaping EVPs that contain SQA and SQE. Aspirated SQE has been shown to cause lipoid pneumonia (Kanaji et al., 2008; Kim et al., 2009; Cha et al., 2018) and has been detected in EVP aerosol emissions (Cowan et al., 2022). Thus, more research is needed to understand the health consequences of chronically inhaling these chemicals as constituents of EVP-produced aerosol. Additional work is also needed to better understand analyte stability over longer periods of time and to further optimize the gas chromatography liner and injection method to increase sensitivity of the method.

## Conclusion

SQE and SQA are readily transmitted from vape products to aerosols that users inhale. Furthermore, accidental inhalation of large amounts of SQE are associated with lipoid pneumonia and many EVALI case patients presented with lipoid pneumonia-like symptoms. Therefore, we developed and validated an analytical method for measuring SQA and SQE in human BAL fluid to see if SQE or SQA were accumulating in EVALI case patient lungs. This rapidly developed method was adequately sensitive, accurate, and precise for the intended purpose of investigating EVP-associated lung injury. The method was applied to BAL fluid samples from both relatively healthy comparators and patients presenting with EVALI. The concentration of SQA and SQE was found to be below the MRL for all BAL fluids. The results from this method helped to focus our public health response away from SQA and SQE and toward the likely primary causal agent, vitamin E acetate. Future application of this method will help to characterize the exposure and health consequences of chronically inhaling SQA and SQE as constituents of EVP-produced aerosol.

## Data Availability

The original contributions presented in the study are included in the article/Supplementary Material, further inquiries can be directed to the corresponding author.
