# Supplementary figures and images for: The Quantitation of Squalene and Squalane in Bronchoalveolar Lavage Fluid Using Gas Chromatography Mass Spectrometry
Source: Front Chem. 2022 Apr 7;10:874373. doi: 10.3389/fchem.2022.874373 (PMC9021504; doi:10.3389/fchem.2022.874373)

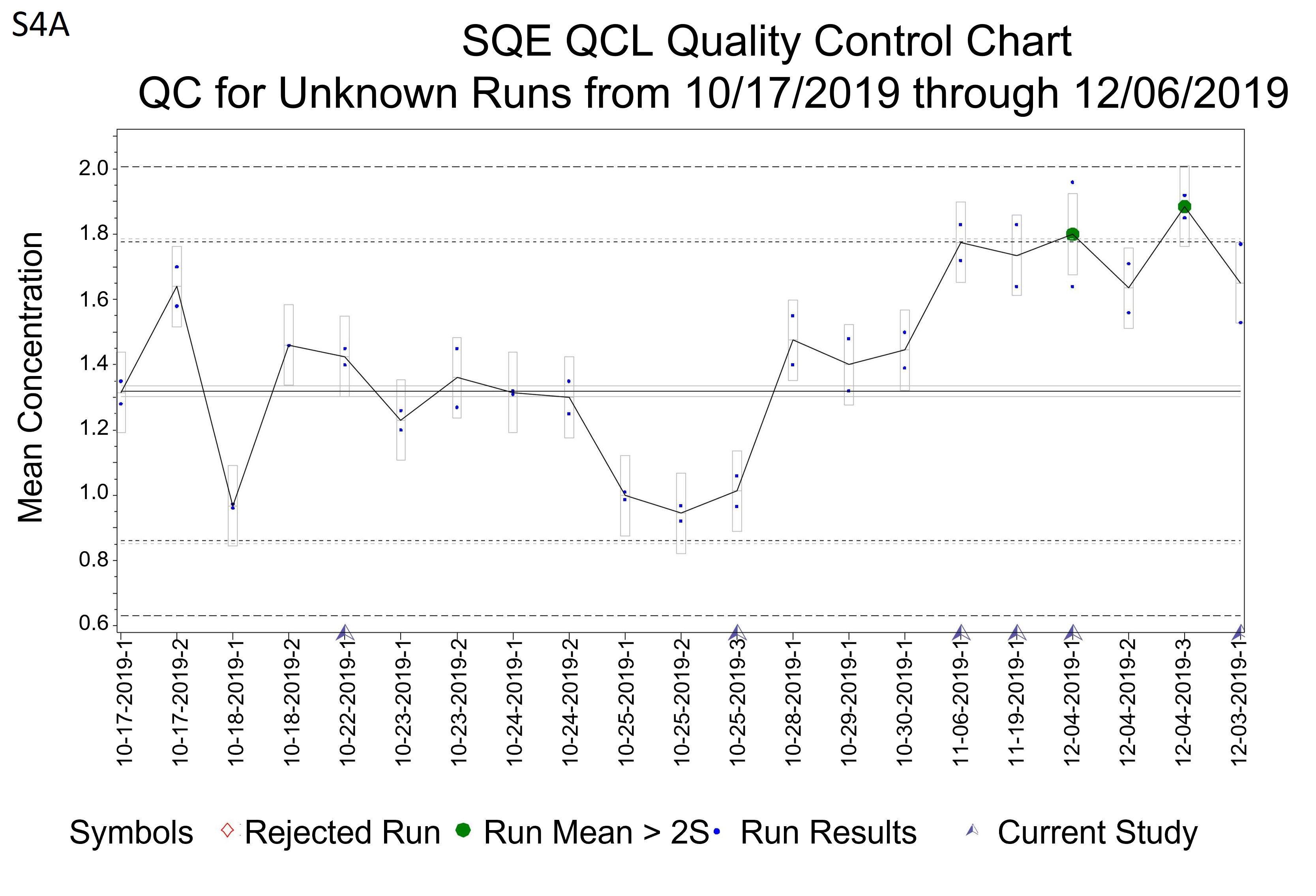

Supplement: Supplementary file 1 [file Image5.png]

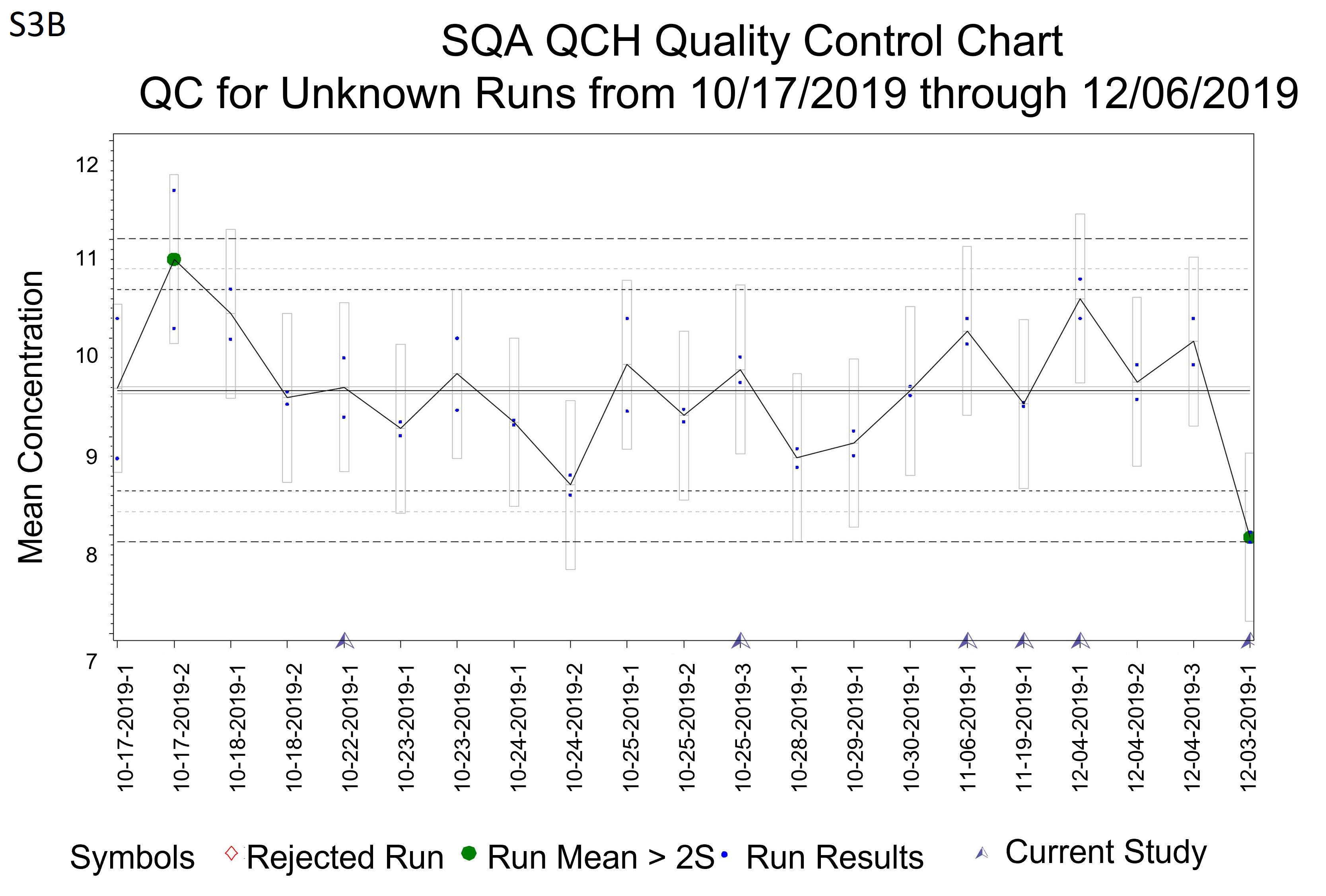

Supplement: Supplementary file 2 [file Image4.png]

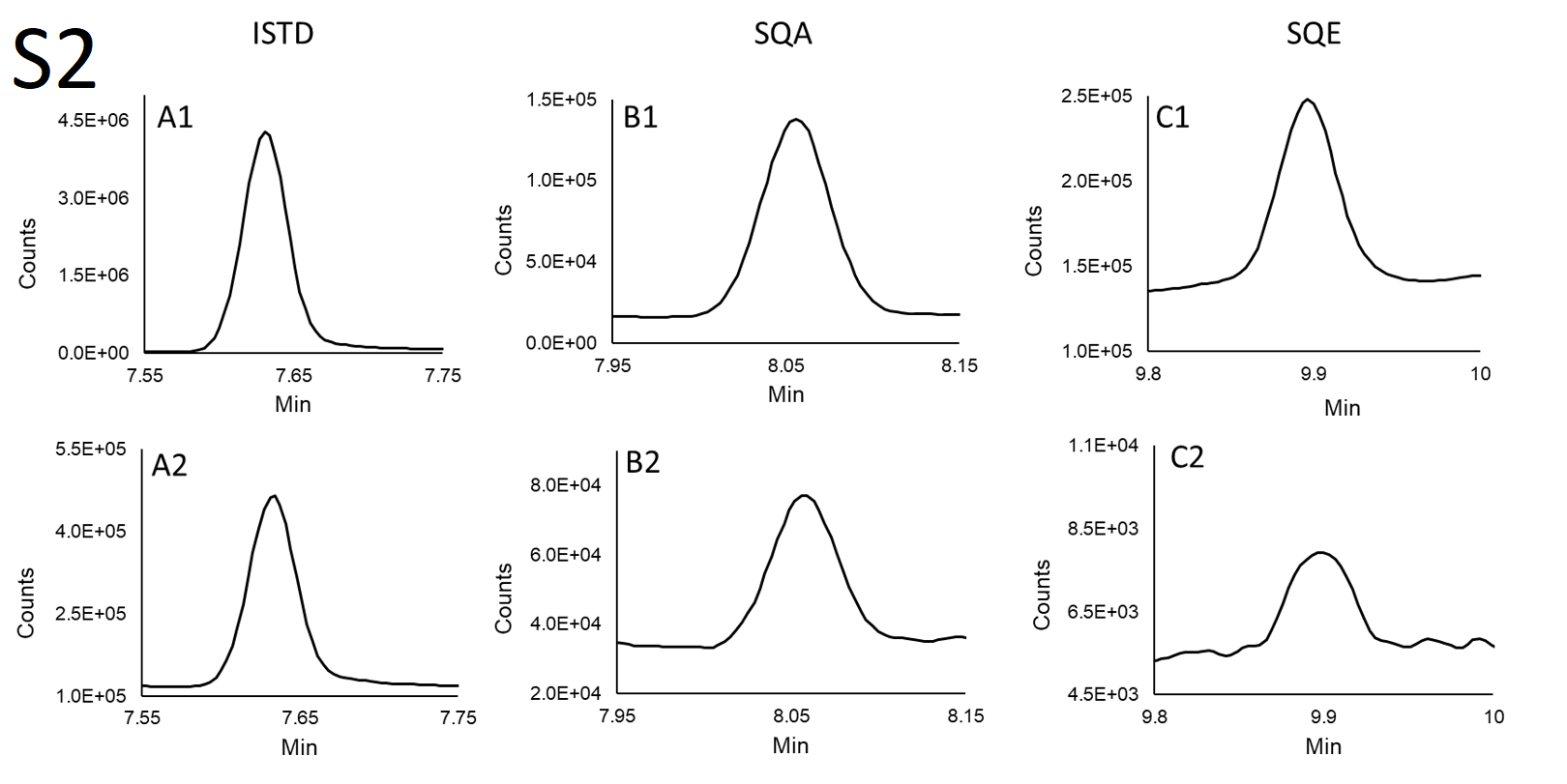

Supplement: Supplementary file 4 [file Image2.png]

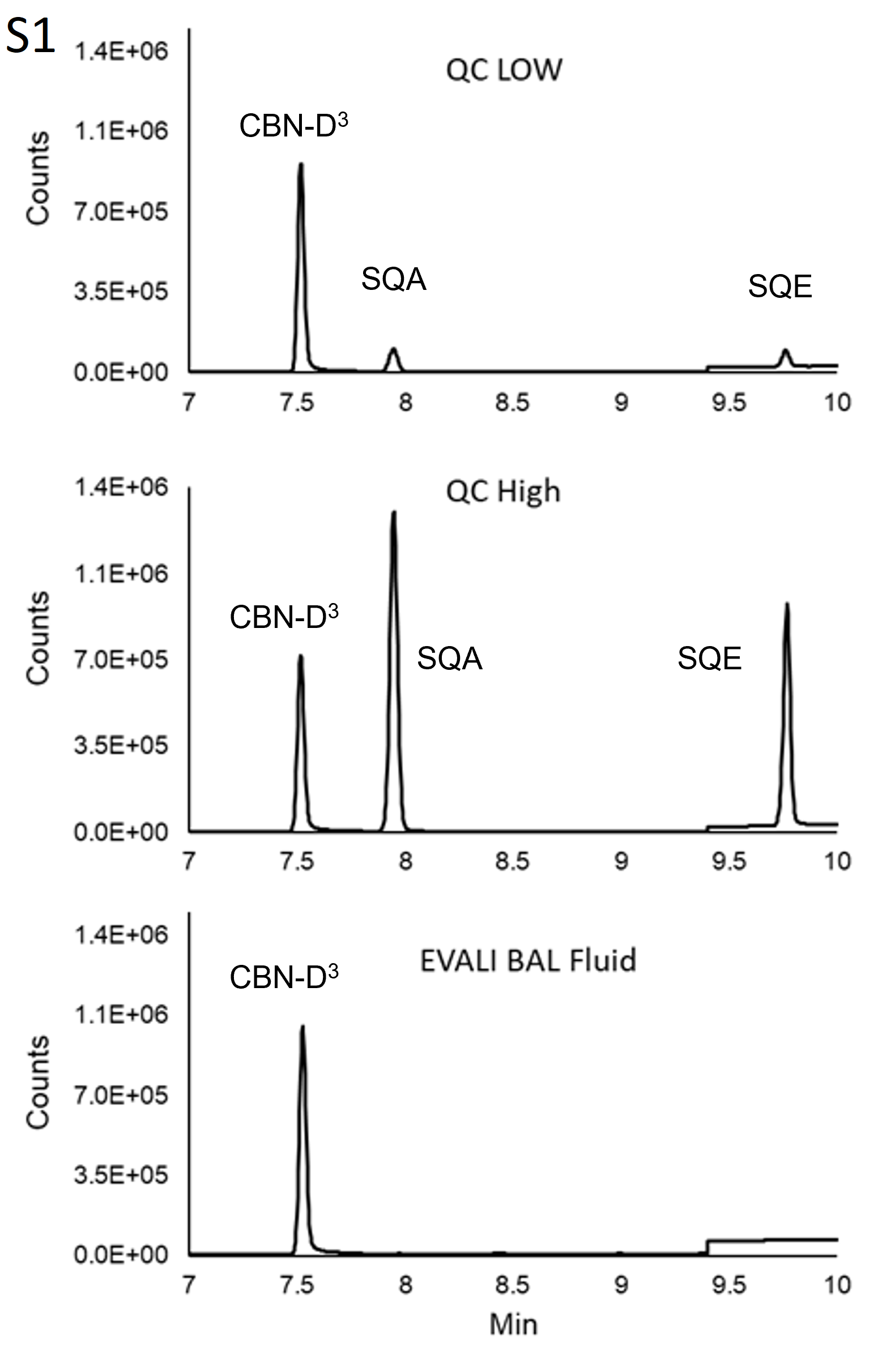

Supplement: Supplementary file 5 [file Image1.png]

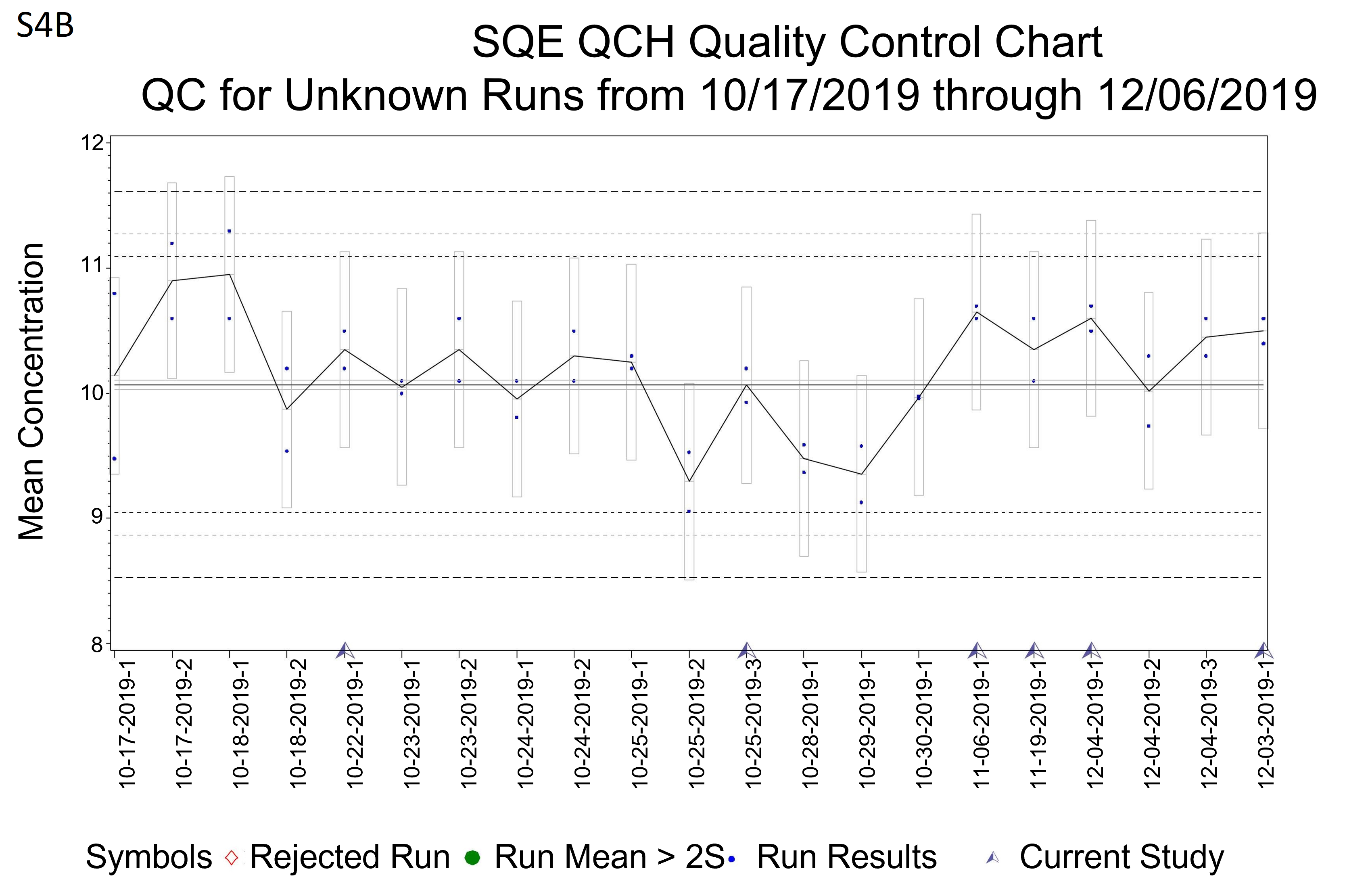

Supplement: Supplementary file 6 [file Image6.png]

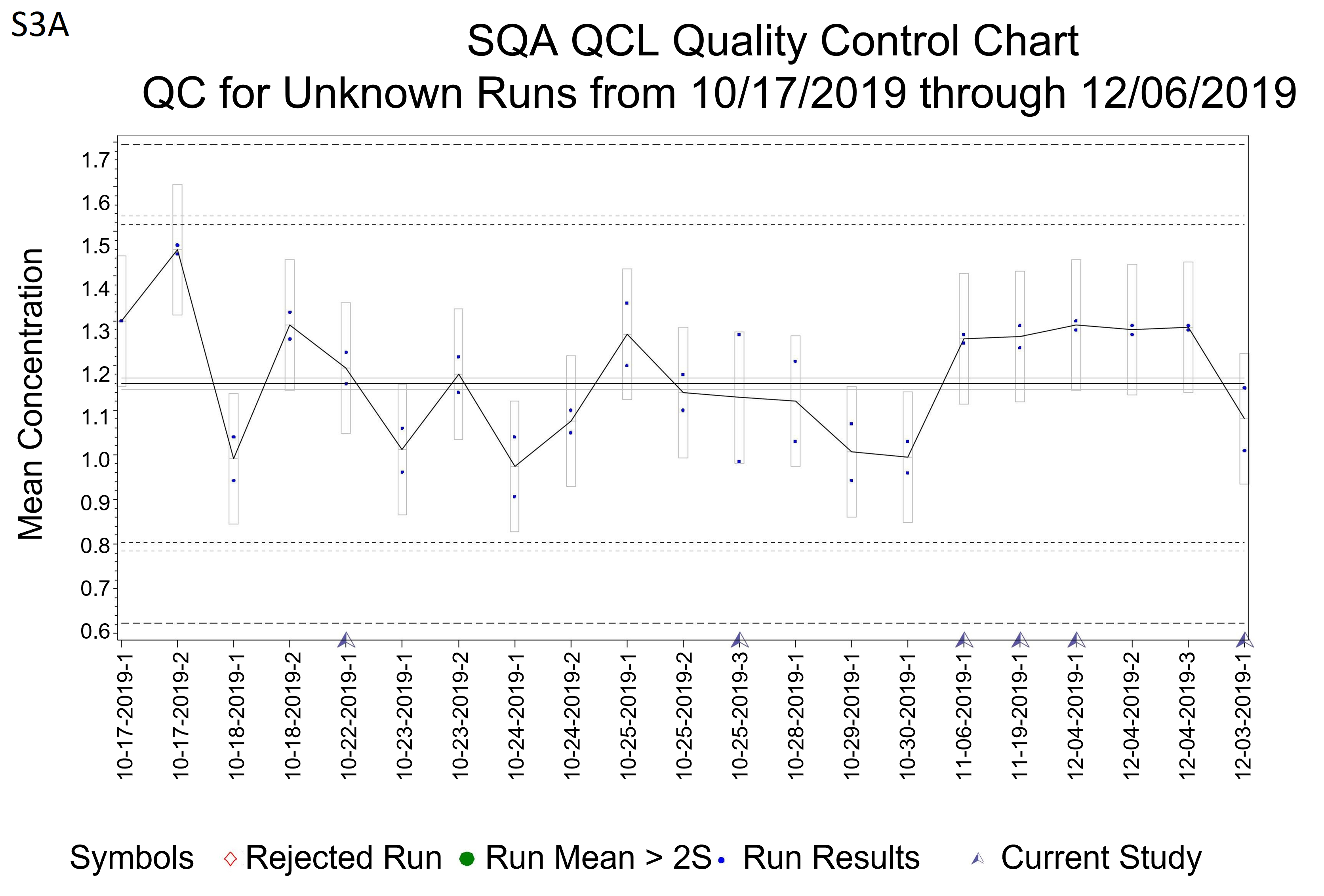

Supplement: Supplementary file 7 [file Image3.png]
