# Supplementary material for: The Quantitation of Squalene and Squalane in Bronchoalveolar Lavage Fluid Using Gas Chromatography Mass Spectrometry
Source: Front Chem. 2022 Apr 7;10:874373. doi: 10.3389/fchem.2022.874373 (PMC9021504; doi:10.3389/fchem.2022.874373)
Supplement: Supplementary file 3 [file DataSheet1.pdf]

Captions

Figure S1. Overlayed extracted ion chromatograms for quantified ions of internal standard and native analytes in a low-level quality control (QCL), a high-level quality control (QCH), and an EVALI patient BAL fluid sample

Figure S2. Extracted ion chromatograms for the quantitation and confirmation ions of CBN-D^3, SQA and SQE: A1 and A2 CBN-D^3 (298 and 313 *m/z*); B1 and B2 SQA (113 and 183 *m/z*) and C1 and C2 SQE (137 and 410 *m/z*). Sample shown is a non-EVALI BAL fluid sample spiked with 1.5 µg/mL.

Figure S3. SQA Shewhart plots of low-level quality control samples (S3A) and high-level quality control samples (S3B) analyzed throughout the duration the BAL fluid study

Figure S4. SQE Shewhart plots of low-level quality control samples (S4A) and high-level quality control samples (S4B) analyzed throughout the duration the BAL fluid study
